# Multiple sessions of liposomal doxorubicin and focused ultrasound mediated blood-brain barrier disruption: safety study

**DOI:** 10.1186/2050-5736-3-S1-P11

**Published:** 2015-06-30

**Authors:** Muna Aryal, Natalia Vykhodtseva, Yong-Zhi Zhang, Nathan McDannold

**Affiliations:** 1 Boston College/Harvard Medical School, Boston, Massachusetts, United States; 2 Brigham & Women’s Hospital/Harvard Medical School, Boston, Massachusetts, United States; 3 Harvard Medical School, Boston, Massachusetts, United States

## Background/introduction

Transcranial MRI-guided focused ultrasound is a rapidly advancing field for delivering therapeutic and imaging agents to the brain. It has the ability to facilitate the passage of therapeutics from the vasculature to the brain parenchyma, which is normally protected by the blood-brain barrier (BBB). Its main advantages are that it is targeted, noninvasive, and readily repeatable in nature. Studies have shown that it can deliver liposomal doxorubicin (DOX), a chemotherapy agent with promise for tumors in the central nervous system, into the brain across BBB. However, prior studies have suggested that this agent may be significantly neurotoxic, even at small concentrations. Here, we studied whether multiple sessions of DOX administered after FUS-induced BBB disruption (FUS-BBBD) caused adverse events in the normal brain. First, we used fluorometry to measure the doxorubicin concentrations in the brain after FUS-BBBD. Next, we performed three weekly sessions with FUS-BBBD ± DOX administration.

## Methods

FUS-BBBD was produced in one hemisphere in 18 male Sprague-Dawley rats (250-350g); the other hemisphere served as a control. Nine animals were assigned to one of two groups: (1) three weekly treatments with FUS and concurrent chemotherapy (FUS+DOX) (N=5), (2) three weekly treatments with FUS only (N = 4). Sonications (0.69 MHz; 0.55-0.81 MPa; 10 ms bursts; 1 Hz PRF; 60s duration) were performed over three weeks in a grid pattern at 5, 9, and 12 targets, respectively, following a schedule described previously in a survival study in glioma-bearing rats (Aryal *et al*., Journal of Control Release, 2013). Each sonication was combined with an i.v. injection of Definity (10μl/kg). DOX (5.67 mg/kg) was administered in fractions before each sonication. Contrast enhanced T1-weighted imaging and T2*-weighted imaging were used to confirm BBBD and detect hemorrhage in the targeted areas, respectively. The animals’ health was monitored regularly, and MRI was obtained to evaluate treatment effects. Seven weeks after the last treatment, animals were sacrificed, and the brains were sectioned and stained for histological analysis. To confirm that we were delivering DOX liposomes across the BBB, nine additional animals were sacrificed four hours after sonication (0.55 MPa; nine targets in a 3×3 grid) and DOX concentrations were measured in both hemispheres (sonicated and non-sonicated) using fluorometry.

## Results and conclusions

We found that clinically-relevant concentrations of doxorubicin (4.8±0.5 μg/g) were delivered to the brain with the sonications parameters, microbubble concentration (Definity, 10 μl/kg), and the administered DOX dose used. We also noted that the resulting concentration was reduced by 38% when the agent was injected 10 minute after the last sonication. In histology, focal planes were unaffected in rats who received FUS-BBBD but not DOX. The brain of one rat who received FUS-BBBD and DOX appeared unaffected at focal plane. Three others had small scars and one had a small infarct. Overall this work demonstrates that ultrasound can deliver clinically-relevant concentrations of DOX across the BBB, particularly when administered before the FUS exposures. The results indicate that while delivery of DOX to the rat brain can result in minor damage, the severe neurotoxicity seen in earlier work does not appear to occur with delivery via FUS-BBB disruption.

